# The impact of severe haemophilia and the presence of target joints on health-related quality-of-life

**DOI:** 10.1186/s12955-018-0908-9

**Published:** 2018-05-02

**Authors:** Jamie O’Hara, Shaun Walsh, Charlotte Camp, Giuseppe Mazza, Liz Carroll, Christina Hoxer, Lars Wilkinson

**Affiliations:** 1 Faculty of Health and Social Care, University of Chester, Chester, UK; 2 HCD Economics, Daresbury, UK; 3 UCL Institute for Liver and Digestive Health, Royal Free Hospital, University College London, London, UK; 4 The Haemophilia Society, London, UK; 5 Novo Nordisk A/S, Vandtårnsvej 114, DK-2860 Søborg, Denmark; 6 HCD Economics, The Innovation Centre, Daresbury, WA4 4FS UK

**Keywords:** Haemophilia, Disease burden, Joint disease, Health-related quality of life, Patient-reported outcome measures

## Abstract

**Background:**

Joint damage remains a major complication associated with haemophilia and is widely accepted as one of the most debilitating symptoms for persons with severe haemophilia. The aim of this study is to describe how complications of haemophilia such as target joints influence health-related quality of life (HRQOL).

**Methods:**

Data on hemophilia patients without inhibitors were drawn from the ‘Cost of Haemophilia across Europe – a Socioeconomic Survey’ (CHESS) study, a cost-of-illness assessment in severe haemophilia A and B across five European countries (France, Germany, Italy, Spain, and the UK). Physicians provided clinical and sociodemographic information for 1285 adult patients, 551 of whom completed corresponding questionnaires, including EQ-5D.

A generalised linear model was developed to investigate the relationship between EQ-5D index score and target joint status (defined in the CHESS study as areas of chronic synovitis), adjusted for patient covariates including socio-demographic characteristics and comorbidities.

**Results:**

Five hundred and fifteen patients (42% of the sample) provided an EQ-5D response; a total of 692 target joints were recorded across the sample. Mean EQ-5D index score for patients with no target joints was 0.875 (standard deviation [SD] 0.179); for patients with one or more target joints, mean index score was 0.731 (SD 0.285). Compared to having no target joints, having one or more target joints was associated with lower index scores (average marginal effect (AME) -0.120; SD 0.0262; *p* < 0.000).

**Conclusions:**

This study found that the presence of chronic synovitis has a significant negative impact on HRQOL for adults with severe haemophilia. Prevention, early diagnosis and treatment of target joints should be an important consideration for clinicians and patients when managing haemophilia.

## Background

Haemophilia is a rare, lifelong bleeding disorder characterised by a deficiency of coagulation factor, with an estimated incidence of the two most common forms (Haemophilia A, a Factor VIII deficiency; and Haemophilia B, a Factor IX deficiency) of 1 per 4000–5000 and 1 per 20,000, respectively [1, 2]. Approximately 70% of cases arise via X-linked recessive inheritance; the remaining 30% arise with no known familial history (‘sporadic’ haemophilia) [3]. Its primary manifestation is prolonged bleeding of the musculoskeletal system and, less frequently, mucosal and cerebral haemorrhages; persons with severe (coagulation factor < 1% of normal) haemophilia are particularly prone to spontaneous bleed events (occurring in the absence of notable trauma).

Approximately 80% of bleed events are intra-articular in nature, two-thirds of which are reported in the knees, elbows, and ankles [4]. Bleed events lead to swelling and acute pain of the affected area, alleviated through infusions of plasma-derived or recombinant coagulation factor and respite care [5]. Frequent bleed events (2–3 in a six-month period) to the same joint site is associated with chronic inflammation of the synovium and reduced joint flexion and mobility, arising as a result of increased volume of fluid within the joint [6]. In the absence of appropriate management, chronic synovitis is a significant risk factor for long-term deterioration of the joint via haemophilic arthropathy, leading to chronic pain, joint disfigurement, and disability [7–9].

With the advent of prophylactic factor concentrate regimens, introduced in Europe in the 1980s, the majority of persons with haemophilia (PWH) aged 30 and under exhibit minimal joint damage relative to that of previous generations [10]. However, risk of inhibitor development, whereby an autoimmune response to factor concentrate renders the product ineffective, is a significant risk for PWH, particularly for newly diagnosed infants in the early days of therapy exposure and those undergoing surgery [11]. In the absence of effective treatment, either with bypass therapies or through ‘training’ the body to accept factor concentrate (‘immune tolerance induction’ or ITI), the presence of an inhibitor can significantly increase bleed frequency and accelerate joint damage [12].

The psychological burden of degenerative, heritable diseases such as haemophilia is an important yet historically overlooked aspect of economic evaluations and health policy considerations, which more often focus on disease-related morbidity and mortality [13]. However, as major therapeutic advances in haemophilia have extended life expectancy and improved clinical outcomes for sufferers, more recent studies have shifted focus to health-related quality of life (HRQOL) as an outcome measure, either with generic or disease-specific instruments [14, 15]. However, the specific impact of musculoskeletal complications of haemophilia has not so far been considered in isolation.

The purpose of this paper is to utilise a recent (2015) observational study in severe haemophilia across five European countries to explore drivers of HRQOL. Specifically, the analysis will explore the relationship between target joints and HRQOL for severe PWH, and the extent to which patient-reported health and wellbeing is driven by long-term clinical outcomes. While this topic has been explored to some detail within single-country studies, the use of data from the ‘Cost of Haemophilia in Europe – a Socioeconomic Survey’ (CHESS) dataset affords access to one of the largest samples of patient-reported HRQOL of recent years.

## Methods

### Data source

CHESS is a retrospective, non-interventional study of severe, inherited haemophilia A and B across five European countries (France, Germany, Italy, Spain, and the UK). The purpose of the study was to generate an annualised economic burden of the condition through reporting of 12-month, haemophilia-related direct and indirect resource use [16]. One hundred and thirty-nine haematologists provided demographic and clinical information for 1285 adult (≥18 years) patients, 541 (42%) of whom completed a corresponding questionnaire covering out-of-pocket medical expenditure, HRQOL (measured using the EQ-5D-3 L) and work loss.

Study exclusion criteria was limited to patients diagnosed with an inhibitor at the time of study capture (*n* = 52), due to a differing risk profile for bleeds and subsequent target joint development among these patients [12, 17].

### EQ-5D

The EQ-5D is one of the most frequently used generic tools for assessing HRQOL [18]. The questionnaire consists of two sections, the first of which (health state) consists of five dimensions (mobility, self-care, usual activities, pain/discomfort, and anxiety/depression), with responses rated on an ordinal (1–3) scale. For each of the five dimensions, the respondent indicates the statement best describing their ‘health state’ at that time, with 1 equivalent to ‘no issues’ and 3 to ‘severe issues’. A health state index ‘utility’ score based on country-specific ‘value sets’ is derived through an amalgam of the five responses, with scores generally ranging from 0 (equivalent to ‘dead’) to 1 (‘perfect health’), though scores of less than zero (states ‘worse than dead’) can also be derived [19].

The second part of the EQ-5D is a visual analogue scale (VAS). The respondent is asked to indicate how good or bad their health is on that day, based on a scale from 0 to 100, where zero represents ‘the worst health’ the respondent can imagine, and 100 ‘the best health state’ the person can imagine. Corresponding country population norms for both the EQ-5D index score and the VAS were used in this analysis, with the exception of Italy, for which the Spanish value set was used.

### Target joint definition

A ‘target joint’ as defined in the CHESS study encompasses any joint with known chronic synovitis; in contrast to previous clinical studies [20], study investigators were given discretion as to how this may be further defined with respect to bleed frequency and period of observation. In order to explore the differential impact of costs associated with lower and upper body joint deterioration, target joints were categorised into two groups based on their location. ‘Upper body’ target joints were those in the shoulders, elbows, wrists, neck, and spine; ‘lower body’ target joints consisted of hips, knees, and ankles. The target joint variable was assessed in three ways: as a binary 0/1 variable; as a binary 0/1 variable split into upper and lower body joints; and as a discrete variable.

### Statistical analysis

Demographic data and EQ-5D index score and VAS responses were compared between the sample of patients with no reported target joints and those with one or more reported target joints. Means were used to describe continuous variables; categorical variables are described as frequencies and proportions. Standard t-tests were conducted in order to test for between-group differences.

The marginal effect of the presence of one or more target joints on EQ-5D-3 L index score was assessed using a generalized linear model (GLM). Health utility data on a ‘poor’-‘good’ scale is often negatively skewed with a large volume of unity values (i.e. perfect HRQOL) and a long ‘tail’ from a select group of less healthy ‘outlier’ patients. The GLM is an extension of the linear regression framework (Eq. 1) suitable for nonparametric outcome variables [21, 22]. The GLM requires a link function relating the conditional mean to the covariates, and a distribution ‘family’ to specify the relationship between the variance and the mean [23]. The log-link function (Eq. 2) in combination with a Poisson distribution (Eq. 3) is frequently used to model EQ-5D index scores and was employed for this analysis [24]. Index scores were transformed to a ‘disutility’ index score (nY = 1-Y) in order to fit the model specification [25, 26]. A confirmatory analysis of the family and link functions was conducted using the modified Park test [21, 22].

1 $$ {NDDCs}_{12 mth}=\alpha +{\beta}_1\left( target\ joints\right)+{\beta}_2{x}_i+\dots +{\beta}_n{x}_n $$

Where *i* = 1, …, *n*

2 $$ E\left[y|x\right]=f\left({x}^{\prime}\beta \right)=\exp \left({x}^{\prime}\beta \right) In\left(E\left[y|x\right]\right)={x}^{\prime}\beta $$

3 $$ y\sim Var\left(y|x\right)\approx {\left(E\left[y|x\right]\right)}^{\lambda } $$

Additional model covariates were country of residence, patient age, and physician-reported presence of mental illness (anxiety and/or depression) via a stepwise inclusion method. Results are presented as average mean effects (AME). All statistical analysis was conducted using Stata 13 [27].

## Results

### Patient characteristics

The average age of the patient cohort was 37.7 years old, with the majority of patients (62.9%) aged between 31 and 40 years (Table 1). Almost two-thirds (61.0%) of patients were receiving therapy via a prophylaxis regimen at the time of the study.

**Table 1:** Patient characteristics (*n* = 515)

Age (mean ± SD)	37.7 ± 15.0
Age categories (%)	
18–30	205 (39.8%)
31–40	119 (62.9%)
41–50	89 (17.3%)
51–60	50 (9.7%)
61 +	52 (10.1%)
Subtype (%)	
Haemophilia A	400 (77.7%)
Haemophilia B	115 (22.3%)
Country (%)	
France	180 (35.0%)
Germany	96 (18.6%)
Italy	118 (22.9%)
Spain	84 (16.3%)
UK	37 (7.2%)
Treatment Strategy (%)	
On-demand	201 (39.0%)
Primary prophylaxis	314 (61.0%)
Physician-reported comorbidities	
Depression	77 (15.0%)
Anxiety	84 (16.5%)
Target joints (total)	714
Target joints (mean ± SD (range))	1.39 ± 1.44 (0–9)
Number of target joints (patient n, %)	
Zero	157 (30.5%)
One	153 (29.7%)
Two	130 (25.2%)
Three or more	75 (14.6%)
Location of target joints (patient n, %)	
Exclusively upper body	92 (25.7%)
Exclusively lower body	188 (52.5%)
Upper and lower body	78 (21.8%)

*Note.* Values are means ± SD or numbers (%)

**Fig. 1:**
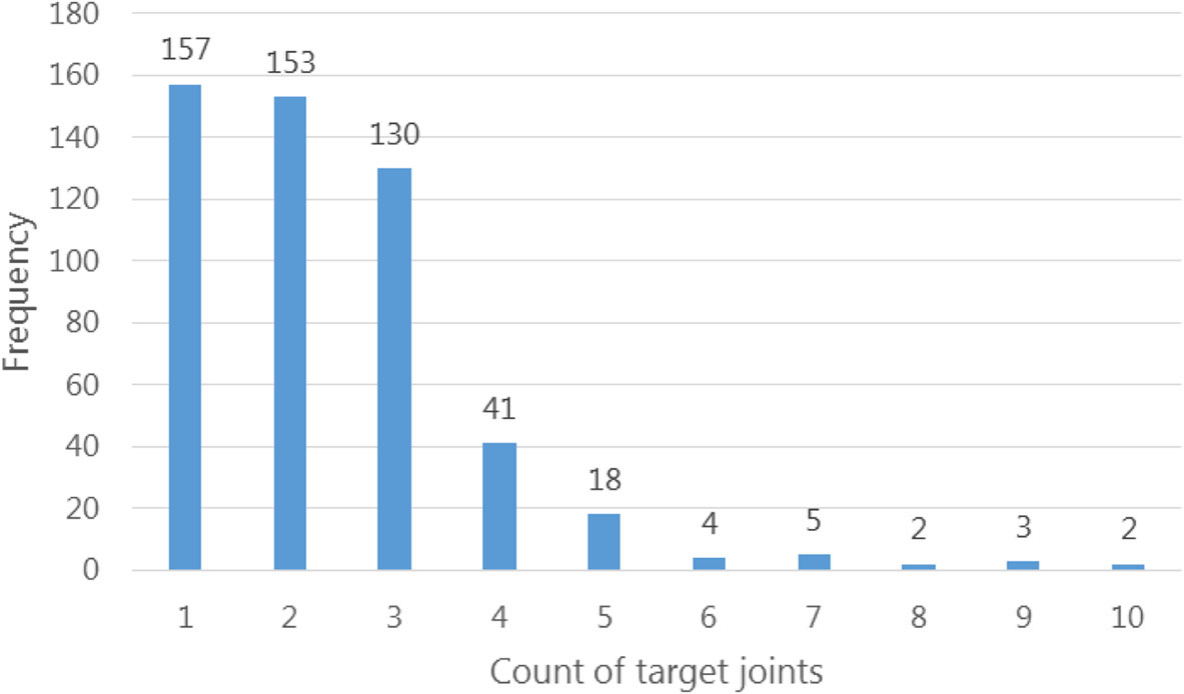
Distribution of study cohort by count of target joints (*N* = 515)

A total of 714 target joints were recorded across the study population (mean 1.39; SD 1.44; range 0–9) (Fig. 1). Three hundred and fifty-eight patients (69.5%) were reported diagnosed with one or more target joints, with the majority (79%) diagnosed with one or two target joints. The majority (52.5%) of patients had target joints exclusively in the lower body.

### Factors influencing HRQOL

The mean EQ-5D index score in the sample was 0.77 (SD 0.27) (Table 2). Age was found to have a negative impact on HRQOL: index scores were found to decrease as patients progressed into each 10-year age cohort. Patients from Germany had the highest index score (mean 0.90; SD 0.12) and the United Kingdom the lowest scores (mean 0.59, SD 0.37). Patients receiving prophylaxis had lower mean index scores compared to on-demand (mean 0.80 versus 0.75).

**Table 2:** EQ-5D-3 L index score by target joint status

	Total (*n* = 515)	No target joints (*n* = 157)	≥1 target joints (*n* = 358)
Total	0.77 ± 0.27	0.87 ± 0.01	0.73 ± 0.29
Age categories			
18–30	0.86 ± 0.01	0.94 ± 0.01	0.82 ± 0.21
31–40	0.78 ± 0.23	0.84 ± 0.16	0.74 ± 0.25
41–50	0.72 ± 0.28	0.86 ± 0.12	0.69 ± 0.30
51–60	0.67 ± 0.34	0.83 ± 0.19	0.63 ± 0.36
61 +	0.61 ± 0.36	0.73 ± 0.31	0.54 ± 0.36
Subtype			
Haemophilia A	0.78 ± 0.26	0.87 ± 0.18	0.74 ± 0.28
Haemophilia B	0.76 ± 0.29	0.86 ± 0.18	0.70 ± 0.32
Country			
France	0.75 ± 0.28	0.87 ± 0.18	0.69 ± 0.30
Germany	0.90 ± 0.12	0.93 ± 0.09	0.88 ± 0.13
Italy	0.85 ± 0.12	0.86 ± 0.14	0.84 ± 0.11
Spain	0.66 ± 0.34	0.71 ± 0.36	0.65 ± 0.33
UK	0.59 ± 0.36	0.78 ± 0.17	0.56 ± 0.38
Treatment strategy			
On-demand	0.80 ± 0.26	0.87 ± 0.21	0.77 ± 0.27
Prophylaxis	0.75 ± 0.01	0.87 ± 0.01	0.71 ± 0.29
Physician reported comorbidities			
Depression	0.60 ± 0.36	0.79 ± 0.33	0.55 ± 0.35
Anxiety	0.80 ± 0.26	0.87 ± 0.21	0.77 ± 0.27
Number of target joints (patient n, %)			
One	–	–	0.76 ± 0.28
Two	–	–	0.76 ± 0.26
Three or more	–	–	0.62 ± 0.31
Location of target joints (patient n, %)			
Exclusively upper body	–	–	0.77 ± 0.27
Exclusively lower body	–	–	0.73 ± 0.28
Upper and lower body	–	–	0.68 ± 0.31

Note: Values are means ± SD

**Fig. 2:**
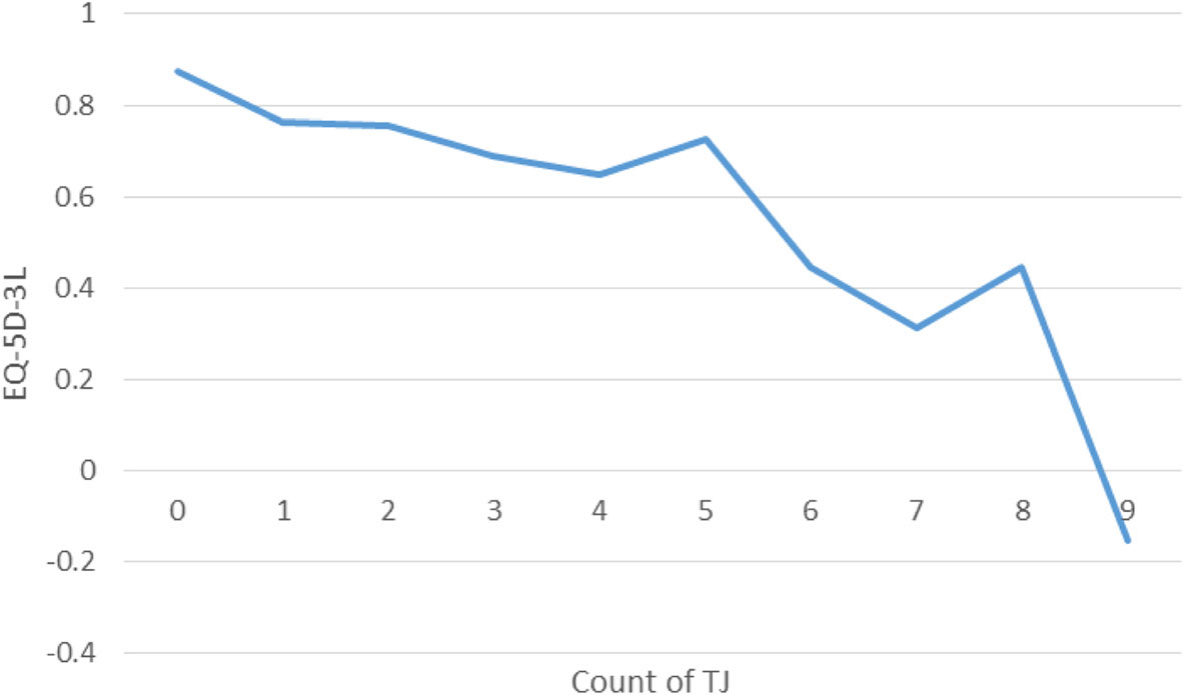
EQ-5D-3 L index score by number of target joints (*N* = 515)

**Fig. 3:**
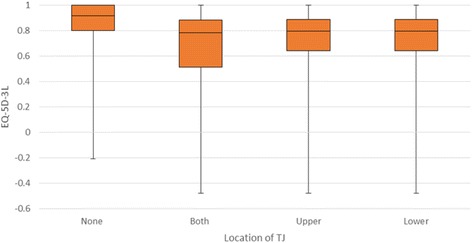
EQ-5D-3 L index score by location of target joint

Patients with no recorded target joints had significantly higher utilities than those with one or more target joints (mean 0.87 versus 0.73), with scores deteriorating as the number of target joints increased (Fig. 2). Index scores among patients with an upper body target joint was broadly similar to those with a lower body target joint (mean 0.77 versus 0.73). Patients with both an upper and lower body target joint had lower index scores versus those with target joints in one location (upper or lower body) (mean index score 0.68, SD 0.31) (Fig. 3).

### Relationship between target joint status and EQ-5D VAS

**Fig. 4:**
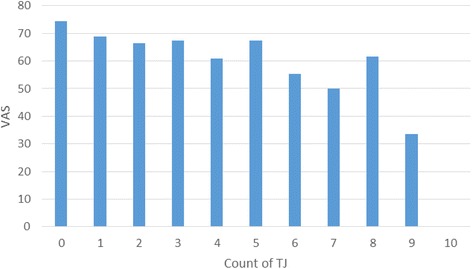
EQ-5D VAS score by count of target joints

Patients with no target joints reported the highest VAS scores (mean 74.3, SD 0.9) (Table 3), while patients with both an upper and lower body target joint reported the lowest VAS scores (mean 64.4, SD 18.3). VAS scores among patients with an upper body target joint were identical to those with a lower body target joint (mean 67.9). Mean reported VAS scores followed a downward trend as the number of target joints increased (Fig. 4).

**Table 3:** EQ-5D VAS score by target joint status

Total	69.3 ± 17.0
Number of target joints (patient n, %)	
Zero	74.3 ± 0.9
One	69.1 ± 15.7
Two	67.1 ± 17.5
Three or more	63.1 ± 16.5
Location of target joints (patient n, %)	
Exclusively upper body	67.9 ± 15.0
Exclusively lower body	67.9 ± 16.7
Upper and lower body	64.4 ± 18.3

Note: Values are means ± SD

### Individual dimensions of the EQ-5D-3 L

Across all five dimensions of the EQ-5D-3 L, the majority of patients reported no problems, with fewer than one in ten patients reporting ‘extreme’ problems in any dimension (Table 4). Across the cohort as a whole, as well as within the cohort of patients with target joints, pain/discomfort and anxiety/depression were the most common dimensions in which ‘extreme’ problems were reported. The cohort of patients with one or more target joints consistently reported a higher frequency of extreme problems compared to those with no target joints: whether that be in mobility (0.0% versus 1.4%); self-care (0.6% versus 2.0%); usual activities (0.6% versus 2.8%); pain/discomfort (1.3% versus 4.7%); and anxiety/depression (3.2% versus 5.6%).

**Table 4:** Frequencies of item responses in each EQ-5D health state dimension by target joint status (%)

	Target joint status		
Dimension	All (*n* = 515)	No target joints (*n* = 157)	≥1 target joints (*n* = 358)
Mobility			
No problems	312 (60.6%)	114 (72.6%)	198 (55.3%)
Some	198 (38.4%)	43 (27.4%)	155 (43.3%)
Extreme	5 (1.0%)	0 (0.0%)	5 (1.4%)
Self-care			
No problems	402 (78.1%)	135 (86%)	267 (74.6%)
Some	105 (20.4%)	21 (13.4%)	84 (23.5%)
Extreme	8 (1.6%)	1 (0.6%)	7 (2.0%)
Usual activities			
No problems	343 (66.6%)	119 (75.8%)	224 (62.6%)
Some	161 (31.3%)	37 (23.6%)	124 (34.6%)
Extreme	11 (2.1%)	1 (0.6%)	10 (2.8%)
Pain/Discomfort			
No problems	205 (39.8%)	95 (60.5%)	110 (30.7%)
Some	291 (56.5%)	60 (38.2%)	231 (64.5%)
Extreme	19 (3.7%)	2 (1.3%)	17 (4.7%)
Anxiety/Depression			
No problems	308 (59.8%)	113 (72.0%)	195 (54.5%)
Some	182 (35.3%)	39 (24.8%)	143 (39.9%)
Extreme	25 (4.9%)	5 (3.2%)	20 (5.6%)

### Multivariate analysis

The presence of one or more target joints was associated with lower EQ-5D index scores (AME -0.120; SD 0.026; *p* < 0.001) (Table 5). Patients in Germany or Italy with one or more target joints had the highest index scores (AME 0.110 and 0.121 respectively; *p* < 0.001); patients in the UK were found to have the lowest mean index scores (AME -0.154, SD 0.060). The AME of patient age on index scores was minimal yet significant (mean 0.005; SD 0.001; p < 0.001). Anxiety/depression was found to be associated with lower index scores (AME -0.106; SD 0.001; p < 0.001).

**Table 5:** Multivariate Poisson regression analyses of disutility derived from EQ-5D index scores

	Model 1	Model 2
Country		
France		Omitted
Germany		-0.110^a^ (0.025)
Italy		-0.121^a^ (0.022)
Spain		0.029^a^ (0.034)
UK		0.154 (0.060)
Age		0.005^b^ (0.001)
Mental illness^c^		0.106^b^ (0.001)
Has target joint	0.173^a^ (0.031)	0.120^a^ (0.026)
AIC	1.002	0.956
BIC	− 3070.013	− 3068.135

Note: Results shown are average mean effect (AME) of each covariate on EQ-5D ‘disutility’ (1 - index score). Standard errors are shown in brackets

^a^Significant at the 95% level ^b^ Significant at the 99% level

^c^Physician-reported anxiety or depression

## Discussion and conclusion

The findings of this analysis suggest an association between musculoskeletal complications of haemophilia and HRQOL: patients with no recorded target joints had the highest mean EQ-5D index and VAS scores; further, the number of target joints was inversely correlated with both measures. There was limited difference in index scores for individuals with either an upper or lower body target joint; patients with target joints in both locations had the lowest HRQOL within both the health state index score and VAS score. The results of the multivariate analysis show that, despite the confounding influences of age and nationality, the presence of one or more target joints remained a significant driver of reduced HRQOL among our study cohort.

The long-term complications of haemophilia can have pronounced effects on HRQOL early in life – due to anxiety over future problems – and later in life, when the complications manifest themselves [28–30]. The analysis supports previous studies which suggest haemophilia patients experience depression and anxiety more often than the general population of a similar age [31]. Fatigue, loss of enjoyment, and reduced leisure pursuits also seem to correlate positively with the presence of target joints [28–30]. The pain and discomfort dimension of the EQ-5D-3 L was found to be particularly sensitive to the presence of target joints, with more than eight times as many patients reporting extreme pain or discomfort when a target joint was recorded.

Our study has several limitations. The definition of a target joint in this analysis differs from current European guidelines that focus on bleed frequency as a measure of target joint status [32]. While the definitions exhibit significant overlap, reporting and observance of a bleed event is more straightforward than obtaining a clinical diagnosis of chronic synovitis, which often requires ultrasound and/or MRI assessment [33]. As discussed elsewhere [34], the definition of a target joint used in the CHESS study encompasses both these standardized definitions based on bleed rates and a consideration for longer-term degenerative changes to the joint tissue and structure arising from repeat bleed events.

Further, while our analysis attempts to create a comprehensive model of the main factors influencing EQ-5D index scores in severe haemophilia, there is the potential that some drivers were not accounted for, such as BMI, HIV/HCV seropositivity, and sociodemographic factors such as employment and marital status [28, 30, 31, 35]. Our choice of HRQOL measure, though used widely, also presents limitations with respect to sensitivity and discriminatory power, particularly in comparison to the more recent five-level (5 L) version [36].

Nevertheless, the results presented in this study suggest that chronic synovitis in severe haemophilia is associated with reduced HRQOL as measured by the EQ-5D. Approaches to minimising the long-term risk of joint damage and deterioration among these patients – beginning at a young age with proactive therapy protocols to minimise bleed frequency and severity – will serve to reduce future psychosocial burdens on patients and are a justification for continued access to preventative therapy protocols. Further studies should seek to combine the cost and HRQOL consequences of bleed events and joint disease, and hence to quantify the cost effectiveness of current therapy protocols for severe haemophilia.

Our analysis demonstrates that haemophilia-related medical complications, such as the presence of one or more target joints, can have a major impact on HRQOL for persons with severe haemophilia.
